# Modulation of lipid biosynthesis by stress in diatoms

**DOI:** 10.1098/rstb.2016.0407

**Published:** 2017-07-17

**Authors:** Olga Sayanova, Virginie Mimouni, Lionel Ulmann, Annick Morant-Manceau, Virginie Pasquet, Benoît Schoefs, Johnathan A. Napier

**Affiliations:** 1 Department of Plant Sciences, Rothamsted Research, Harpenden AL5 2JQ, UK; 2 Metabolism, Bioengineering of Microalgal Molecules and Applications, Mer Molécules Santé, UBL, IUML—FR 3473 CNRS, University of Le Mans, Le Mans-Laval, France

**Keywords:** diatoms, lipids, omega-3, nutrition, biofuel, stress

## Abstract

Diatoms are responsible for up to 40% of the carbon fixation in our oceans. The fixed carbon is moved through carbon metabolism towards the synthesis of organic molecules that are consumed through interlocking foodwebs, and this process is strongly impacted by the abiotic environment. However, it has become evident that diatoms can be used as ‘platform’ organisms for the production of high valuable bio-products such as lipids, pigments and carbohydrates where stress conditions can be used to direct carbon metabolism towards the commercial production of these compounds. In the first section of this review, some aspects of carbon metabolism in diatoms and how it is impacted by environmental factors are briefly described. The second section is focused on the biosynthesis of lipids and in particular omega-3 long-chain polyunsaturated fatty acids and how low temperature stress impacts on the production of these compounds. In a third section, we review the recent advances in bioengineering for lipid production. Finally, we discuss new perspectives for designing strains for the sustainable production of high-value lipids.

This article is part of the themed issue ‘The peculiar carbon metabolism in diatoms’.

## 1. Diatoms and the use of carbon in their metabolism

### (a) Availability of carbon in marine environment

Inorganic carbon (Ci) in the atmosphere has dramatically increased during the industrial era to exceed 400 ppm CO_2_ in 2016, whereas it did not exceed 300 ppm before the industrial revolution [1]. CO_2_ dissolution and biological carbon fixation (the so-called ‘biological pump’) make the oceans the main Ci sink on Earth. The former mechanism depends on atmospheric CO_2_ pressure, temperature, salinity and pH. At the usual pH of seawater, around 8.0–8.3, the dissolved inorganic carbon (DIC) is mainly in the form of 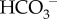, so that the equilibrium concentration of CO_2_ ranges between 10 and 20 µM [2,3]. Air-equilibrated seawater contains some 180 times more ionic Ci (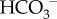 and 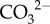) than CO_2_ [4]. Altogether, the oceans have taken up approximately 30% of the anthropogenic CO_2_ emissions causing ocean pH decrease and temperature increase.

The biological pump relies on carbon fixation by photosynthetic organisms living freely or in symbiosis. However, CO_2_ concentration in seawater is low regarding the affinity of ribulose-1,5-bisphosphate carboxylase/oxygenase (Rubisco), the key enzyme of CO_2_ fixation. Indeed, the half-saturation constant of Rubisco measured in diatoms ranges between 30 and 40 µM [5]. To supply efficiently Rubisco, some marine diatoms, as well as other microalgae, can take up both CO_2_ and 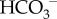 from seawater and concentrate CO_2_ close to Rubisco [6,7] using CO_2_-concentrating mechanisms (CCM) involving different carbonic anhydrase isoforms [8–12]. Moreover, a C_4_-like pathway highlighted in few diatom species could play a role in CCM [13–15]. Altogether, about half of the Earth's primary production takes place in unicellular microalgae [16]. Diatoms contribute up to 20% of global carbon fixation and thus play a major ecological role as primary producers [17–19]. When these organisms die, they settle on the seabed where carbon is sequestrated for geological timescales and might ultimately result in fossil fuels.

### (b) Regulation of carbon metabolism: the influence of inorganic carbon availability

All the environmental factors such as temperature, irradiance levels and chemical compounds (heavy metals and nutrients) influence carbon metabolism. In this section, only the influence of inorganic carbon availability on diatom metabolism will be discussed. Regardless of the Ci form taken up by diatoms, Ci is finally reduced into carbohydrates during photosynthesis. These molecules are necessary for cell growth, respiration and synthesis of the other cell organic compounds (e.g. [20]). Diatoms export triose phosphates from the plastids and, under nutrient-replete conditions, store them in cytosolic vacuoles as the β-1,3-glucan polymer, namely chrysolaminarin [21,22] during the day, catabolizing this in the dark [23]. In diatoms living in microphytobenthic communities, carbohydrates largely contribute to the production of extracellular polymeric substances [24]. Changes of the environmental parameters that impact photosynthesis trigger modifications of the relative activity of the different metabolic pathways, resulting in lipid accumulation [25] that can increase the cell's buoyancy [22,26]. Caballero *et al*. [23] observed little change in chrysolaminarin accumulation or consumption during nitrogen starvation in *Phaeodactylum tricornutum*. Song *et al*. [27] demonstrated that gas flow rate in the culture medium plays an important role in *P. tricornutum* growth and production of lipid that is mostly composed of saturated and monounsaturated fatty acids (MUFA). When the 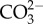/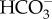 ratio increases to 0.125, the content of C16 : 1 increases to the highest value of 57%. The effects of CO_2_ concentration on C : N : P ratio in six diatoms show different trends according to the species and the light regime. In the range of dissolved CO_2_ from 1.5 to 37.7 µmol kg^−1^, either C : N and C : P ratios stay almost constant in *P. tricornutum*, decrease as in *Thalassiosira weisflogii* or increase in *Amphora glacialis* [28]. A CO_2_-dependent decrease in growth rate was observed at dissolved CO_2_ concentration below 10 µmol kg^−1^. Boelen *et al*. [29] reported that the photophysiological performance of the marine Antarctic diatom *Chaetoceros brevis* is not significantly affected by a pCO_2_ ranging from 190 to 750 ppmv under different irradiance regimes.

Although it is usually considered that diatoms are not mixotrophic [30], it has been reported that repeated additions of organic substrate such as urea [31] or acetate [32] can sustain mixotrophic growth of *P. tricornutum* UTEX-640 and *Skeletonema costatum*, respectively. Light intensity and quality impact on pigment contents, photosynthesis and nutritional value of diatoms [33,34], with photosynthesis/irradiance curves commonly used to characterize photoacclimation [35–37]. Generally, benthic diatoms have a lower maximum net photosynthesis but a higher light utilization coefficient than planktonic species [10,38]. Because chlorophyll *a* and carotenoid contents vary according to light environment and structural rearrangement inside chloroplasts [33,39], the motile benthic diatom *Navicula* cf. *recens* uses motility to select the optimal light exposure according to its photophysiological preferences.

## 2. Lipid accumulation and omega-3 long-chain polyunsaturated fatty acid biosynthesis in diatoms

### (a) Glycerolipid biosynthesis

**Figure 1.**
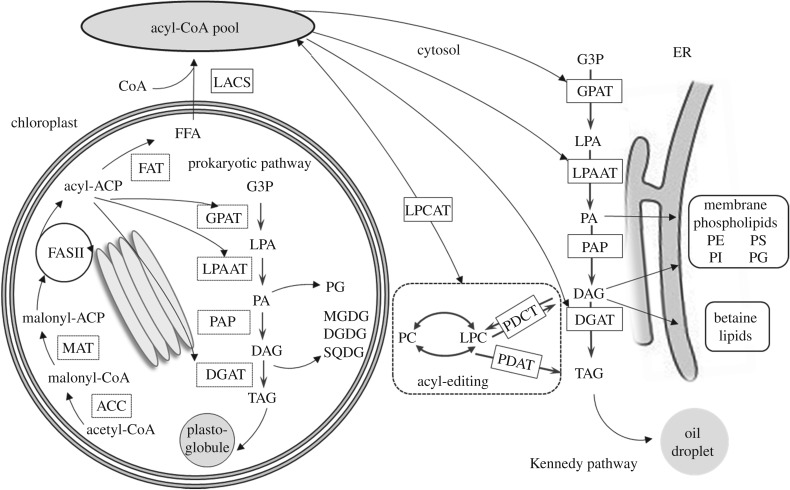
Schematic representation of lipid biosynthesis in microalgae. ACC, acetyl-CoA carboxylase; DAG, diacylglycerol; DGAT, diacylglycerol acyltransferase; DGDG, digalactosyldiacylglycerol; ER, endoplasmic reticulum; FAT, fatty acyl-ACP thioesterase; G3P, glycerol-3-phosphate; GPAT, glycerol-3-phosphate acyltransferase; LACS, long-chain acyl-CoA synthase; LPAAT, lysophosphatidic acid acyltransferase; LPA, lysophosphatidic acid; LPCAT, lysophosphatidylcholine acyltransferase; MAT, malonyltransferase; MGDG, monogalactosyldiacylglycerol; PA, phosphatidic acid; PAP, phosphatidic acid phosphatase; PC, phosphatidylcholine; PDAT, phospholipid diacylglycerol acyltransferase, PDCT, phosphocholine transferase; PE, phosphatidylethanolamine; PG, phosphatidylglycerol; PI, phosphatidylinositol; PS, phosphatidylserine; SQDG, sulfoquinovosyldiacylglycerol; TAG, triacylglycerol.

Many microalgae accumulate triacylglycerols (TAGs) in response to changes in environmental conditions. Depending on the cultivation conditions, some oleaginous species of diatoms have the ability to produce lipids up to 40–60% of the biomass and synthesize high content of highly valuable omega-3 long-chain polyunsaturated fatty acids (LC-PUFAs) such as eicosapentaenoic acid (20 : 5Δ^5,8,11,14,17^, EPA) and docosahexaenoic acid (22 : 6Δ^4,7,10,13,16,19^, DHA) (table 1). However, the mechanisms of the high lipid production in these organisms remain unclear. Fatty acid and glycerolipid biosynthesis have been studied in detail in the model plant *Arabidopsis thaliana* [51]. Similar pathways are predicted to occur in diatoms [52]. In photosynthetic organisms, the production of LC-PUFAs begins with de novo fatty acid synthesis in the plastids [53]. The first committed step is the formation of malonyl-CoA following the carboxylation of acetyl-CoA, catalysed by acetyl-CoA carboxylase (ACCase), a multi-subunit enzyme found in most prokaryotes and in the chloroplasts of plants and algae. The malonyl-CoA produced from the carboxylation of acetyl-CoA by ACCase is the central carbon donor for fatty acid synthesis. The fatty acid synthase (FAS) multi-subunit complex catalyses the production of C16 : 0 and C18 : 0 fatty acids (figure 1). Before entering the fatty acid synthesis cycle, the malonyl group of malonyl-CoA is transferred by malonyltransferase (MAT) to the cofactor acyl carrier protein (ACP) to form malonyl-ACP, which then undergoes a series of condensation reactions with acetyl-CoA, catalysed by a β-ketoacyl-ACP synthase (KAS) [51]. The candidate activities involved in fatty acid biosynthesis in diatoms have not yet been functionally characterized.

**Table 1.** Oil content (% of dry mass) and EPA/DHA content (% of total fatty acids) of selected diatom species. DHA, docosahexaenoic acid; EPA, eicosapentaenoic acid; ND, not detected.

species	total lipids	EPA	DHA	references
*Amphora exigua*	33–45	5–13	ND	[40]
*Chaetoceros muelleri*	33.6	12.8	0.8	[41,42]
*Fistulifera solaris*	48–60	5–20	—	[43,44]
*Melosira nummuloides*	33	6.5–12	3.9–11	[40]
*Navicula lyra*	37–42	5.5–9.3	<1	[40]
*Nitzschia laevis*	25–69	19.1		[45]
*Phaeodactylum tricornutum*	20–30	34.5	<1	[46,47]
*Seminavis gracilenta*	35–43	11–16	<1	[48]
*Skeletonema costatum*	21.1	29.2	3.1	[41,42]
*Thalassiosira pseudonana*	21–31	12.2	3.4	[49,50]

Each FAS cycle of reactions leads to the extension of the fatty acid chain by two carbon atoms, usually ending with the synthesis of saturated C16 : 0- and C18 : 0-ACPs. These acyl groups are then either retained in the plastids where they are transferred by chloroplastic acyltransferase to glycerol-3-phosphate (G3P) for incorporation into organellar membrane lipids (the so-called ‘prokaryotic’ pathway) or are hydrolysed from ACP by specific fatty acyl-ACP thioesterases (FATs) that release free fatty acids (FFA) in the inner envelope of the chloroplast. Some FATs can also release fatty acids containing upto 14 carbons. Alternatively, C16 : 0- and C18 : 0-ACPs can be desaturated by soluble palmitoyl- or/and stearoyl-ACP desaturases and subsequently released by FATs. These FFA are exported to the cytosol and are esterified to coenzyme A (CoA) by a long-chain acyl-CoA synthase (LACS) located in the outer envelope of the plastid to form acyl-CoAs [54]. The precise mechanism for this export step in algae is unknown and requires further investigation. By analogy with higher plants, it is predicted that these extraplastidic acyl-CoA esters are then transferred to endoplasmic reticulum (ER) or acylated onto a phospholipid backbone by the action of lysophosphatidylcholine acyltransferase (LPCAT) where they can undergo modifications before further participation in the synthesis of membrane lipids or storage TAGs (‘eukaryotic’ pathway).

In chloroplasts, the prokaryotic pathway generates PA and DAG and lipids such as galactolipids (monogalactosyldiacylglycerol, MGDG, and digalactosyldiacylglycerol, DGDG), sulfoquinovosyldiacylglycerol (SQDG) and phosphatidylglycerol (PG) [52,55]. Lipids synthesized in the chloroplast are characterized by the presence of a C16 acyl group at the *sn*-2 position of the glycerol backbone. In diatoms, the chloroplast is assumed to have originated from a secondary endosymbiosis event [52] and is surrounded by four membranes, representing a possible continuum between the ER and the outermost membrane of the plastid [56]. Therefore, the precise localization of the lipids synthesized via the prokaryotic pathway is difficult to predict.

In eukaryotes, TAG synthesis occurs via the Kennedy pathway [57] through the stepwise esterification of G3P and occurs in the microsomal fraction, presumptively the ER. The transfer of a fatty acid from the acyl-CoA pool to the *sn*-1 position of G3P is the first committed step in this pathway and is catalysed by glycerol-3-phosphate acyltransferase (GPAT), resulting in the production of lysophosphatidic acid (LPA). In plants and algae, the situation is further complicated by the presence of two types of GPAT, i.e. chloroplastic GPAT and extra-plastidial GPAT. Lysophosphatidic acid acyltransferase (LPAAT) catalyses the esterification of LPA at the *sn*-2 position of the glycerol backbone, giving rise to phosphatidic acid (PA).

Dephosphorylation at the *sn*-3 position of PA by phosphatidic acid phosphatase (PAP) generates diacylglycerol (DAG). Both PA and DAG are precursors for TAG and membrane phospholipids (mainly phosphatidylcholine, PC, and phosphatidylethanolamine, PE) biosynthesis. Diatoms also synthesize non-phosphorus glycerolipids, the so-called betaine lipids (BL), that are not found in higher plants [58]. BLs contain a polar amino-acyl group linked by an ether bond to the *sn*-3 position of the glycerol backbone. These lipids are presumably located in extra-plastidial membranes and may be involved in the transfer of fatty acids from the cytoplasm to the chloroplast. Comprehensive study of the glycerolipid content of *P. tricornutum* revealed the presence of diacylglyceryl hydroxymethyltrimethyl-β-alanine (DGTA), which has been found in some other algae [59]. Together with PC, it represents the major lipids present in the extra-plastidial membranes of *P. tricornutum.*

In plant seeds, large amounts of unsaturated fatty acids are synthesized on PC [60]. PC can undergo an acyl-editing cycle (the eponymous Lands cycle), by hydrolysis of the fatty acid at the *sn*-2 position, followed by a re-acylation with a fatty acid derived from the acyl-CoA pool [61]. Polar head exchanges can also occur by the action of a PC-DAG phosphocholine transferase (PDCT) [60]. In many plants, PC acyl editing and phosphocholine headgroup exchange between PC and DAGs control the majority of acyl fluxes through PC to provide PUFA for TAG synthesis [62]. It is unclear whether the same PC turnover occurs in diatoms, and given the abundance of BL, if that lipid also might act as either an acyl donor or receiver for this process.

The final step of the Kennedy pathway for the production of TAG is the acylation of 1-2-DAG on the *sn*-3 positions which can be achieved either with an acyl-CoA, catalysed by diacylglycerol acyltransferase (DGAT) or by transfer of an acyl-chain from PC by phospholipid : diacylglycerol acyltransferase (PDAT). Two types of DGAT isoforms, DGAT1 and DGAT2, are membrane-bound acyltransferases and play a major role in TAG biosynthesis. Most eukaryotic organisms contain a single copy of each DGAT gene, while photosynthetic microalgae appear to have multiple copies of DGAT2 genes: four putative DGAT2 genes have been identified in the diatom *Fragilariopsis cylindrus* and five isoforms have been found in *P. tricornutum* [63]. Accumulation of LC-PUFAs in glycerolipids has been studied in *P. tricornutum* [59] and *Fistulifera solaris* [64]. Complete characterization of *P. tricornutum* glycerolipidome in low-phosphate and low-nitrogen conditions demonstrated that the production of TAG most likely relied on a DAG substrate synthesized via the Kennedy pathway and on the combined activity of a DGAT and a PDAT adding a third acyl-group at position *sn*-3 [59]. A similar mechanism for EPA incorporation into TAG was observed in *Fi. solaris*, suggesting that EPA was desaturated on PC and the PC-based acyl-editing and headgroup exchange processes may contribute to the EPA incorporation into TAG [64].

Major activities of TAG biosynthesis have been annotated and partially characterized in several diatom genome sequences [65–67]. However, the precise subcellular localization of the diatom enzymes involved in fatty acid and glycerolipid biosynthesis continues to require further investigation.

### (b) Biosynthesis of polyunsaturated fatty acids

**Figure 2.**
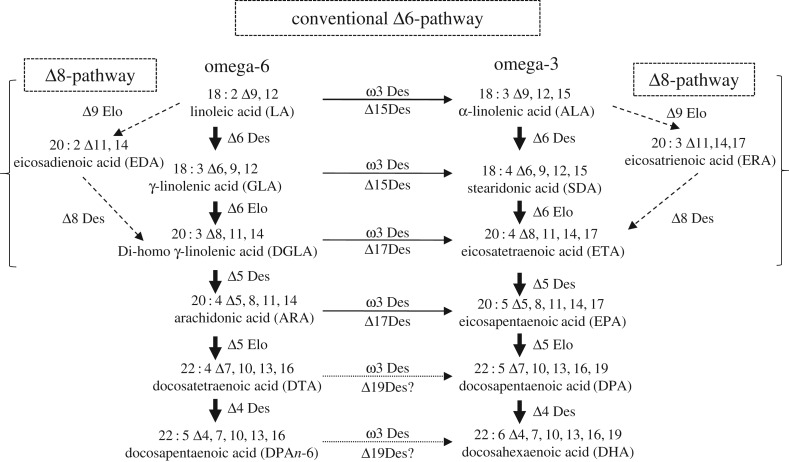
The biosynthesis of LC-PUFAs in diatoms. Schematic representation of Δ6- and Δ8-pathways for LC-PUFAs biosynthesis.

The saturated fatty acids (C16 : 0 and C18 : 0) produced de novo can undergo further desaturation by plastidic or ER desaturases. Desaturation of C18 : 0 can be catalysed either by the soluble stearoyl-ACP Δ9-desaturase (SAD) of the chloroplast stroma [68] or by an extra-plastidic ER-bound acyl-CoA Δ9-desaturase (ADS) [69], generating oleic acid (OA, 18 : 1Δ9). OA becomes a substrate for further desaturation by Δ12-desaturase resulting in the production of linoleic acid (LA; 18 : 2Δ9,12) which subsequently may be converted into α-linolenic (ALA; 18 : 3Δ9,12,15) by the action of Δ15-desaturase (figure 2).

Linoleic acid and α-linolenic acid are essential fatty acids (EFA) and serve as the precursors for omega-6 and omega-3 LC-PUFAs, respectively [70]. The biosynthesis of LC-PUFA from LA and ALA may follow two converging routes: the predominant, or ‘conventional’ Δ6-pathway operating in most PUFA-synthesizing organisms, and an ‘alternative’ Δ8-pathway. To produce LC-PUFAs, a series of alternating desaturation and elongation steps are required. The ‘conventional’ pathway starts with Δ6-desaturation of both LA and ALA, resulting in the synthesis of γ-linolenic acid (GLA, 18 : 3 Δ6,9,12) and stearidonic acid (SDA, 18 : 4 Δ6,9,12,15), respectively. These two Δ6-desaturated fatty acids are then chain-elongated by two carbons via a Δ6-specific elongase which only accepts C18 fatty acids containing a Δ6 double bond, to yield di-homo γ-linolenic acid (DGLA; 20 : 3 Δ8,11,14) and eicosatetraenoic acid (ETA; 20 : 4 Δ8,11,14,17). A second desaturation, via the Δ5-desaturase, then produces arachidonic acid (ARA; 20 : 4 Δ5,8,11,14) and EPA.

Some marine organisms and protists use an ‘alternative’ Δ8-pathway in which LA and ALA are elongated by the action of a Δ9-specific elongase, yielding eicosadienoic acid (EDA; 20 : 2 Δ11,14) and eicosatrienoic acid (ETrA; 20 : 3 Δ11,14,17), prior to two sequential desaturations by a Δ8- and Δ5-desaturases to generate ARA and EPA, similar to the conventional pathway.

In DHA-accumulating microbes, the conversion of EPA to DHA may be carried out by the ‘microbial’ linear desaturation-elongation pathway [71]. In diatoms, EPA is most likely elongated by a specific Δ5-elongase to docosapentaenoic acid (DPA; 22 : 5 Δ7,10,13,16,19) which is then desaturated by a Δ4-specific desaturase to yield DHA.

All the corresponding genes encoding putative desaturases and elongases have been identified in diatoms and the presence of a highly active ‘conventional’ Δ6-pathway has been demonstrated for several diatom species such as *P. tricornutum* [72–75], *T*. *pseudonana* [76], *F. cylindrus* [77], *Nitzchia* sp. [78] and *Fi. solaris* [67]. Functional characterization of candidate activities involved in EPA and DHA biosynthesis from *P. tricornutum* and *T. pseudonana* indicates that these have the capacity to use both omega-6 and omega-3 intermediates to synthesize EPA and DHA [73,74,76].

LC-PUFA biosynthesis has been well studied in the model diatom *P. tricornutum*, in which the majority of EPA accumulates in polar lipids, especially in galactolipids such as MGDG and DGDG [59,79,80]. *In vivo* labelling in *P. tricornutum* revealed that EPA can apparently be synthesized by a number of different routes, involving Δ6- and Δ8-pathways with the predominant route proceeding via Δ6-desaturation of LA and using intermediates of ω6- and ω3-pathways [81]. Both Δ6- and Δ5-desaturation [73] and Δ6-elongation take place in the ER, and newly synthesized EPA is then imported into the plastids for incorporation into galactolipids [52,55]. Studies of the heterologous expression of *P. tricornutum* desaturases in yeast indicate that these are phospholipid-dependent activities (contrary to LC-PUFA elongases that use acyl-CoA as substrates), providing evidence that the substrate-dichotomy issue also exists in diatoms [72,82]. As the *P. tricornutum* lipidome contains only minor amounts of fatty acid intermediates of the EPA biosynthetic pathway, the accumulation of EPA as a single end-product is highly efficient in this diatom. Although *P. tricornutum* synthesizes small amounts of DHA and the putative gene encoding a Δ4-desaturase has been identified through sequence comparison, the activity of this candidate desaturase has yet to be confirmed. The absence of sequences associated with the ‘alternative’ Δ8-pathway may indicate that some of the activities associated with the conventional pathway also have (under some circumstances) the capacity to accept alternative substrates.

Similar sets of genes for LC-PUFA biosynthesis (i.e. Δ9-, Δ12-, Δ6-, Δ5-, ω3-Δ4 desaturases and Δ6-elongase) have been identified in the genomes of *T. pseudonana* [76], *Fi. solaris* (with the exception of Δ4 desaturase) [67] and in *Nitzchia* sp. transcriptome [78].

One unusual feature of the predicted pathways for LC-PUFA biosynthesis in two species of diatoms, *P. tricornutum* and *Fi. solaris*, is the apparent absence of predicted ω3-desaturases localized in the ER [55,83]. Such enzymes, including Δ15-, Δ17- and hypothetical Δ19-desaturases, convert ω6-fatty acids into their ω3-counterparts [84], and are considered to be essential for the enrichment of ω3-fatty acids over ω6 forms. In genome sequences for both these diatoms, only a single candidate gene for a plastidial ω3-desaturase has been identified. This raises the possibility of a dual localization of these putative plastidial ω3-desaturases, with access to both plastidial and ER-located substrates, similar to that found in *Chlamydomonas reinhardtii* [85].

### (c) Regulation of eicosapentaenoic acid synthesis

During the past decade, all the primary activities required for the biosynthesis of LC-PUFA (elongases and desaturases) have been identified from multiple different species, and omega-3 biosynthesis has been successfully reconstituted in plants and microorganisms [86,87]. During PUFA synthesis, regulation occurs as a consequence of numerous factors [88] and environmental factors such as nutrients, light or temperature [89].

#### (i) Influence of carbon availability

A carbon source is absolutely required for lipid and fatty acid accumulation. Indeed, without this source, independently of nutrient deprivation, biomass or lipid synthesis is not possible. Diatoms, being photoautotrophic eukaryotes, use Ci such as CO_2_ as their carbon source and light as their energy source, thus participating in the reduction of atmospheric CO_2_ [20,90]. Bicarbonate has been shown to increase lipid accumulation in *P. tricornutum* [91]. This effect does not result from bicarbonate addition *per se* but from the concomitant pH increase [92]. Using ^13^C labelling, an equal incorporation of ^13^C carbon from bicarbonate has been observed in the C16 fatty acid chains incorporated into TAGs and PC, indicating that *P. tricornutum* is able to assimilate similar amounts of carbon regardless of nitrate availability. This suggests that an other internal lipid pool such as BL could be involved in TAG or PC synthesis [93].

In *P. tricornutum*, high levels of CO_2_ (0.15%) induce an increase in LC-PUFA levels, representing more than 16% of dry cell mass by comparison with around 7 and 8% obtained, respectively, with low (0.015%) and mid-CO_2_ (atmospheric, 0.035%) cultured cells [88]. The EPA content represents about 11.6% dry cell mass with high CO_2_ levels and 5–7% of dry cell mass under low and mid-CO_2_ conditions. However, the influence of CO_2_ concentration depends on the studied diatom species and specifically, the levels of EPA in the diatom concerned. Notably, *P*. *tricornutum* is rich in EPA and, consequently, levels of CO_2_ enhance EPA productivity. By contrast, *Chaetoceros muelleri* with low PUFA content [94] has no change in its EPA content when CO_2_ concentrations vary from 0.03 to 30% [95].

Mixotrophy with glycerol as a carbon source and ammonium as a nitrogen source enhances growth of *P. tricornutum*. The periodic supplementation of culture medium with glycerol and ammonium chloride increased the productivity of EPA by a factor of 10 when compared with photoautotrophically control conditions [96] (see also Finazzi [97]).

#### (ii) Influence of other nutrients

In response to salt stress, diatoms are able to change their lipid and fatty acid compositions to modify their membrane permeability. For example, *Nitzschia laevis*, Chen *et al.* [98] by testing different salt concentrations (from 10 to 30 g l^−1^) in *Nitzschia laevis*, have shown that the highest levels of EPA were obtained with a salt concentration of 20 g l^−1^, in parallel with the highest level of galactolipids and phospholipids, with EPA representing more than 70% of the total fatty acids in these polar fractions. These changes in the unsaturation of membrane lipids were suggested to be related to a modification of membrane permeability and fluidity under salt concentration as an acclimation to salinity stress.

Among nitrogen sources that can be used by diatoms, NaNO_3_, NH_4_Cl and urea have been tested in *Cylindrotheca fusiformis* and *Cylindrotheca closterium*. Whatever the nitrogen source, EPA levels were not modified, even though the maximum lipid accumulation was observed with NH_4_Cl in both species [99].

#### (iii) Environmental factors

Environmental factors have different effects on lipid and fatty acid production in *P. tricornutum*. Salinity, nitrogen concentration, light intensity or temperature can act differently on fatty acid composition and total fatty acid content. Indeed, with a salinity from 15 to 35 ppt or with different irradiance levels (50–150 µmol m^−2^ s^−1^), no changes in EPA levels have been observed after 7 days of growth. Decreased levels of EPA were observed under nitrogen limitation conditions (1.24 versus 12.35–49.40 mg l^−1^) or increased temperature (25°C versus 15°C) [100].

*Light*. In the marine diatom *S. costatum*, the highest amounts of EPA are obtained at the end of the logarithmic growth phase and during the stationary phase under high light levels (saturating irradiance) [32].

The quality and strength of UV irradiance also has distinct effects on fatty acid composition in diatoms. It has been reported that in benthic diatoms, even if modifications of fatty acid composition can occur after long-term (30 days) exposure to UV treatments, the UV-A exposure induces an increase in saturated fatty acids, while the UV-B exposure increased the unsaturated fatty acid (EPA) levels. Lipid content was increased after a short-term (6 h) UV-B exposure [101].

*Temperature*. Low temperature impacts lipid synthesis. For instance, when *P. tricornutum* is grown at 22°C for 8 days and then shifted to 10°C, the percentage of lipids increased [89] (table 2). Within 24 h after the temperature shift, a 61% increase was observed (table 2), suggesting that the mechanisms involved in the acclimation to low temperature are very rapid. The relative amount of EPA was modified in response to decreased temperature: it was increased by more than 17% within 48 h after the temperature shift and reached 38% after 4 days of thermal stress (table 2). The EPA enrichment can be even further increased by supplementing the growth medium with nitrogen and phosphate [89]. Similar results have been obtained with *Odontella aurita*. This diatom is used in aquaculture and in human nutrition [102]. In this taxon, the levels of DHA were also higher than when the diatoms were grown at room temperature [103].

**Table 2.** Total lipid content (% of biomass) and EPA distribution (% of total fatty acids) in control (22°C) and stressed cultures (10°C) of *P. tricornutum* after 1, 2, 4 and 8 days of temperature stress. For the methods used for lipid extraction and analysis, see the electronic supplementary material, SD1. After a one-way ANOVA used in analysis of the influence of temperature stress, SNK multiple comparison test results are arranged in increasing order from left to right: *a* < *b* < *c* (*p* < 0.05).

			EPA level	
day	stress temperature (°C)	lipid content (% biomass)	total lipids	galactolipids
1	22	7.8±1.3^*a*^	32.1±1.6^*a*^	40.2±1.8^*ab*^
	10	14.3±1.8^*c*^	31.6±2.3^*a*^	42.4±2.2^*b*^
2	22	8.2±0.6^*a*^	32.9±1.4^*a*^	39.4±1.1^*ab*^
	10	7.4±0.9^*a*^	38.7±2.1^*b*^	50.9±2.5^*c*^
4	22	8.0±2.1^*a*^	29.6±2.9^*a*^	39.1±1.8^*ab*^
	10	9.3±1.1^*ab*^	40.7±0.2^*b*^	50.0±0.21^*c*^
8	22	10.7±0.7^*b*^	33.1±1.6^*a*^	37.1±4.3^*a*^
	10	9.4±1.3^*ab*^	40.6±3.0^*b*^	50.1±6.2^*c*^

## 3. Bioengineering in diatoms

Advances in next-generation sequencing technology and genetic transformation techniques together with transcriptomics, proteomics and metabolomics studies have greatly contributed to algal research. During the past few years, nuclear genomes of the pennate diatoms *P. tricornutum*, *Fi. solaris* and *F. cylindrus* and the centric diatom *T. pseudonana* have been sequenced, providing insights into the genome evolution of these distinctive groups [65–67,104]. Based on genomic and transcriptomic data, several metabolic networks for lipids have been constructed and PUFA biosynthetic pathways elucidated in the oleaginous diatoms *P. tricornutum*, *Fi. solaris* and *Nitzschia* sp. [55,56,67,78,83,105].

Recently, the first proteomic analysis of the oil-body-associated proteins in a diatom has been reported [106]. This study revealed several protein candidates associated with the oil bodies in oleaginous strain *Fi. solaris* and demonstrated that a proteomic approach may provide new insights into TAG accumulation in oil bodies.

Various genetic transformation systems have been developed in diatoms and several LC-PUFA synthesizing species have been successfully transformed by microparticle bombardment (*Chaetoceros* sp. [107], *Cyclotella cryptica* and *Navicula saprophila* [108], *C. fusiformis* [109,110], *Fistulifera* sp. [111], *P*. *tricornutum* [112,113], *T*. *pseudonana* [110]) and electroporation (*P. tricornutum* [114–116] and *Chaetoceros gracilis* [117]).

Several recent studies have demonstrated the advantages of a genetic engineering approach to increase the oil content and manipulate fatty acid composition in different species of diatoms (table 3). A number of strategies could be employed to regulate genes involved in lipid metabolism and LC-PUFAs biosynthesis.

**Table 3.** Recent advances in metabolic engineering of diatoms to increase lipid accumulation and production of LC-PUFAs.

approach	gene	species	outcome	references
overexpression	GPAT	*P. tricornutum*	increase in neutral lipid content and FA	[118]
	FA elongases	*T. pseudonana*	1.4-fold increase in EPA, 4.5-fold increase in DHA	[119]
	Δ5-elongase	*P. tricornutum*	8-fold increase in DHA	[120]
	malic enzyme	*P. tricornutum*	2.5-fold increase in lipid content	[121]
	GPDH	*P. tricornutum*	increase (60%) in neutral lipid content and MUFAs	[122]
	Δ5-desaturase	*P. tricornutum*	increase in neutral lipid content and FA	[123]
	DGAT2	*P. tricornutum*	increase in neutral lipid content	[124]
	acyl-ACP thioesterase	*P. tricornutum*	increased saturated fatty acids	[125]
	thioesterase	*P. tricornutum*	increased FA content up to 72%	[126]
	ACCase	*C. cryptica*, *N. saprophila*	no change in lipid content, increased (2–3×) ACC activity	[127]
silencing	UGPase	*P. tricornutum*	increase in lipid content	[128]
	nitrate reductase	*P. tricornutum*	43% increase in lipid content	[129]
	pyruvate dehydrogenase kinase (PDK)	*P. tricornutum*	increase (80%) in neutral lipid content	[130]
	multi-functional lipase/phospholipase/acyltransferase	*T. pseudonana*	increased lipid yields without affecting growth	[131]
targeted genome modification	meganucleases/TALENs disruption of UDP-glucose pyrophosphorylase gene	*P. tricornutum*	45-fold increase in triacylglycerol accumulation	[132]

During nuclear transformation, transgenes are integrated randomly, making targeting of specific genes more complicated. Rapid development of genome-editing techniques has greatly improved the potential for genetic engineering of microalgae. Targeted and stable modification of the *P. tricornutum* genome through disruption of UDP-glucose pyrophosphorylase has been achieved by using both meganucleases and transcription activator-like effector nucleases (TALENs) [132]. The mutant diatom produced more lipid (45-fold increase in TAG accumulation) than the control when grown in nutrient-stressed media. This generation of an enhanced lipid-producing strain demonstrates the power of genome engineering.

A similar successful example of using TALEN technology has been reported by Weyman *et al*. [133] who used TALENs to interrupt the gene encoding the urease enzyme in *P. tricornutum.* Metabolomic analysis revealed a build-up of urea, arginine and ornithine in the urease knockout lines. This technique allowed clarification of the role of urease in the urea cycle and also improved the molecular toolkit for diatom engineering. Other mechanisms for the targeted knockdown/out of endogenous genes include RNA silencing using RNAi and antisense RNA techniques [134], and high-throughput artificial-micro RNA (armiRNA) techniques [135].

Most recently, a highly efficient CRISPR/Cas9-based system has been developed to create stable targeted gene knockouts in *P. tricornutum* [136]. The reported genome editing method will allow cost-effective functional studies in microalgae and ultimately the engineering of specific traits.

Another valuable contribution to the development of novel tools for algal biotechnology is based on the introduction of an episome via conjugation into the cells of *P. tricornutum* and *T. pseudonana* [137]. A large DNA fragment has been delivered and maintained, suggesting that this new approach could facilitate metabolic engineering of entire biosynthetic pathways in diatoms.

### (a) Overexpressing activities of fatty acid and triacylglycerol biosynthetic pathways

One of the first examples of direct targeting of lipid biosynthesis was the overexpression of an ACCase from the diatom *Cyc. cryptica* [127]. Despite a two to threefold increase in ACCase activity, no significant enhancement in lipid production in transgenic cells was observed. Based on these findings, it was considered that overexpression of ACC enzyme alone might not be sufficient to enhance the whole lipid biosynthesis pathway. One explanation that has been suggested is that under nitrogen stress, the build-up of precursors to the ACCases may play a more significant role in TAG synthesis than the actual ACCase levels [138].

Malic enzyme is involved in pyruvate metabolism and carbon fixation and should be taken into consideration when engineering microalgal strains with enhanced oil content. Overexpression of *P. tricornutum* malic enzyme (PtME) resulted in markedly increased lipid production in transgenic cells [135]. The total lipid content was enhanced 2.5-fold and reached 57.8% of dry cell mass with a growth rate similar to that of wild-type. The neutral lipid content was further increased by 31% under nitrogen-deprivation conditions. This study demonstrated a significant increase in the oil content in the transgenic diatom, suggesting a new target for developing microalgal strains for industrial production.

Another study demonstrated the important role of glycerol-3-phosphate dehydrogenase (GPDH) in lipid biosynthesis [136]. Overexpression of GPDH in *P. tricornutum* resulted in a 6.8-fold increase of the glycerol concentration per cell compared with the wild-type, indicating that the overexpression of GPDH promoted the conversion of dihydroxyacetone phosphate (DHAP) to G3P. There was a 60% increase in neutral lipid content, reaching 39.7% of dry cell mass in transgenic cells in the stationary phase, despite a 20% decrease in cell concentration. Fatty acid profiling showed that the levels of C16- and C18-MUFA significantly increased.

GPAT is the critical enzyme that catalyses the first step of TAG formation. A GPAT has been identified and functionally characterized in *P. tricornutum* [118]. Significant differences in lipid content and fatty acid composition were detected between transgenic and wild-type cells. The neutral lipid content determined by Nile red fluorescence staining was increased twofold. Fatty acid composition showed a significantly higher proportion of unsaturated fatty acids compared with wild-type. EPA levels increased by 40%, while those of C16 : 0 and MUFA decreased by 45% and 12%, respectively. These results suggest that the identified GPAT could efficiently upregulate TAG and biosynthesis in *P. tricornutum*.

Another approach included the direct modification of fatty acid composition through the expression of acyl-ACP thioesterases to control the chain length. Acyl-ACP thioesterases hydrolyse the acyl moiety of the fatty acid from acyl-ACP, releasing FFA. Heterologous expression of plant acyl-ACP thioesterases specific towards the generation of short-chain lauric acid (C12 : 0) and myristic acid (C14 : 0) in *P. tricornutum* resulted in an increased accumulation of shorter chain length fatty acids [125]. Similarly, overexpression of the acyl-ACP thioesterases with specificity for C18 fatty acids may improve LC-PUFA synthesis.

A putative thioesterase from *P. tricornutum* (PtTE; *Phart2_33198*) has been characterized and heterologously expressed in *Escherichia coli*, demonstrating moderate activity for C18 : 1-ACP and low activity for saturated fatty acids (C16 : 0 and C18 : 0) [126]. Overexpression of PtTE in *P. tricornutum* resulted in an almost twofold increase in both C16 : 0 and C16 : 1 and only a minor increase in C18 : 1 and EPA, enhancing the total fatty acid content to 72%.

Overexpression of acyltransferases could represent another valuable strategy for manipulation of lipid content and fatty acid composition in microalgae. An isoform of type 2 diacylglycerol acyltransferase (DGAT2), which participates in TAG assembly has been identified from diatom *P. tricornutum* [124]. Overexpression of DGAT2A in *P. tricornutum* resulted in the production of oil bodies, with a 35% increase of neutral lipids measured by Nile red fluorescence staining and a significant increase of EPA. The growth rate of transgenic microalgae remained similar, maintaining a high biomass. However, only one transgenic clone has been analysed and the role of DGAT2A in lipid enhancement requires further investigation.

Overexpression of biosynthetic enzymes of the LC-PUFA pathway may provide an effective way to improve the EPA and DHA content in marine microalgae. The first successful attempt in engineering the omega-3 trait in transgenic algae was reported recently on the overexpression of heterologous genes in *P. tricornutum* [120]. *Phaeodactylum tricornutum* can accumulate EPA to levels up to 35% of total fatty acid content, representing a good source for the industrial production of this valuable omega-3 LC-PUFA. However, *P*. *tricornutum* does not naturally accumulate significant levels of DHA. Heterologous expression of the *Ostreococcus tauri* C20 Δ5-elongase in *P. tricornutum* resulted in up to eightfold increase in DHA, and this level has been further increased with the co-expression of the acyl-CoA-dependent Δ6-desaturase, when compared with wild-type strains [120]. Importantly, DHA was shown to accumulate in TAGs, with several new TAG species detected in the transgenic strain. No detrimental effects to *P. tricornutum* growth rates were observed in transgenic cells: they were comparable to that of wild-type strains. This is the first report of enhancing the levels of omega-3 LC-PUFAs in a diatom by iterative metabolic engineering. This successful example indicates that metabolic engineering of diatoms represents a promising strategy for optimizing accumulation of beneficial omega-3 LC-PUFAs.

**Figure 3.**
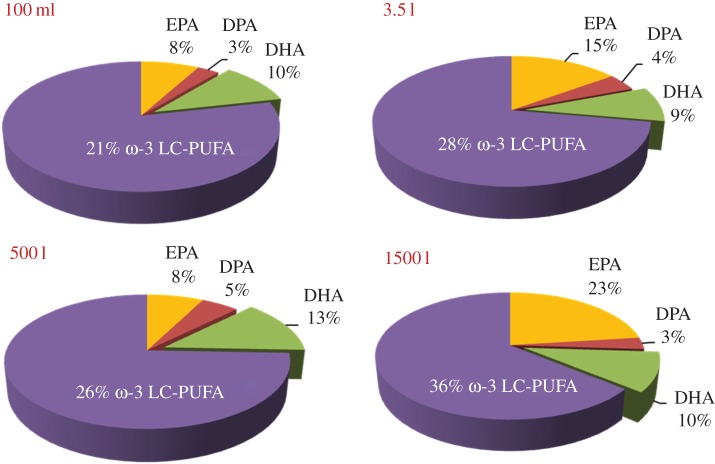
Omega-3 LC-PUFA accumulation in transgenic strain Pt_Elo5 grown in 100 ml flask, a 3.5 l bubble column, a 550 l closed photobioreactor and a 1500 l raceway pond.

This transgenic strain of *P. tricornutum* (Pt_Elo5) has been further investigated for the scalable production of EPA and DHA [139]. Detailed studies were carried out on the effect of different media, carbon sources and illumination on omega-3 LC-PUFAs production by transgenic strain Pt_Elo5 grown in a 3.5 l bubble column, a 550 l closed photobioreactor and a 1500 l raceway pond with artificial illumination. In addition to ambient light, ponds were supplemented by halogen bulbs, light-emitting diode strips and fluorescent tubes (light level up to 110 µmol photons m^−2^ s^−1^) which were housed under polythene sheeting for culture/environment protection. A significant production of DHA over EPA has been observed in cells grown in a photobioreactor, equivalent to 2.1 mg l^−1^ DHA in a mid-exponentially growing algal culture. Omega-3 LC-PUFAs production in a raceway pond was an impressive 36% of TFA, averaging 23% EPA and 10% DHA (figure 3). These results clearly demonstrate the efficacy of large-scale cultivation of the transgenic Pt_Elo5 and is development as a platform for the industrial production of EPA and DHA.

When the Δ5-desaturase gene *PtD5b* was cloned and overexpressed in *P. tricornutum*, fatty acid composition was altered [123]. The neutral lipid content determined by Nile red fluorescence staining indicated an increase in transgenic cells. Engineered cells showed a similar growth rate to with the wild-type, thus keeping high biomass productivity.

A recent study has demonstrated that overexpression of endogenous fatty acid elongase genes in *T. pseudonana* can increase levels of EPA and DHA up to 1.4- and 4.5-fold, respectively [119].

### (b) Blocking competing pathways

An alternative strategy to increase lipid accumulation is to block competing pathways to enhance the metabolic flux channelling to TAG biosynthesis.

Starch is a major carbon and energy storage compound in many microalgae. In diatoms, the fixed carbon is stored as the β-1,3-glucan, known as chrysolaminarin. This is a water-soluble polysaccharide stored in vacuoles. In diatoms, chrysolaminarin synthesis shares common carbon precursors with lipid synthesis. Thus, shunting carbon precursors from the chrysolaminarin synthesis pathway is an efficient way to boost oil accumulation [140]. UDP-glucose serves as the substrate for chrysolaminarin biosynthesis [141]. The effects of the suppression of UDP-glucose pyrophosphorylase (UGPase) on chrysolaminarin biosynthesis and carbon allocation in *P. tricornutum* were investigated in two studies*.* Daboussi *et al*. [132] reported a 45-fold increase in TAG accumulation in the transgenic strains, generated through the disruption of the UDP-glucose pyrophosphorylase using TALEN. A complementary study demonstrated that silencing of UDP-glucose pyrophosphorylase resulted in significant decreases in chrysolaminarin content and increases in lipid synthesis [128]. These findings suggest that UGPase is a rate-limiting enzyme and may play an important role in chrysolaminarin biosynthesis and carbon allocation. The results confirmed the feasibility of increasing lipid production through redirecting photosynthetically assimilated carbon away from starch synthesis to neutral lipid synthesis.

The pyruvate dehydrogenase complex (PDC) catalyses the conversion of pyruvate into acetyl-CoA, NADH and CO_2_, thus directing the carbon flow into the TCA cycle. PDC activity indicates the rate of the entry of pyruvate into the TCA cycle and subsequently to oxidative phosphorylation or fatty acid synthesis. PDC activity is primarily regulated by reversible phosphorylation by PDK, which phosphorylates and deactivates *PtPDK* [142]. Ma *et al*. [130] have identified a putative PDK gene (PtPDK) from *P. tricornutum* and generated a *PtPDK* antisense knockdown transgenic diatom. Neutral lipid content determined by Nile red staining of transgenic cells increased up to 82%, although the fatty acid composition was not altered. Interestingly, the transgenic cells showed slightly lower growth rate but similar to the wild-type cell size.

When starved for nutrients, microalgae redirect carbon towards biosynthesis of storage lipids, TAGs. Another strategy to develop an efficient pathway to enhance lipid production is by suppressing a diatom's ability to incorporate nitrogen. Levitan *et al*. [129] have studied the effect of nitrogen stress on the remodelling process in *P. tricornutum* by knocking down the gene encoding for nitrate reductase, a key enzyme required for the assimilation of nitrate. The transgenic strains exhibited 40–50% of the mRNA, protein content and enzymatic activity of the wild-type, concomitant with a 43% increase in cellular lipid content. This study indicated that a nitrate reductase knocked-down strain shunted approximately 40% more carbon towards TAGs than the wild-type without losing photosynthetic capacity, suggesting that redirecting carbon towards lipid biosynthesis can be achieved by altering the expression of genes distinct from those directly involved with lipid biosynthesis.

An alternative strategy to increase lipid accumulation is to decrease lipid catabolism. The targeted knockdown of lipid catabolism, and specifically lipases, could potentially improve the accumulation of FFA and enhance lipid content with less impact on the primary carbon pathways associated with growth. Trentacoste *et al*. [131] using antisense RNAi constructs successfully engineered *T. pseudonana* to disrupt lipid catabolism by targeting an enzyme with lipase activity. This targeted knockdown resulted in an increase in the accumulation of TAG and total lipid yield without impacting growth rate, under both continuous light and alternating light/dark, continuous growth and nutrient replete versus nutrient deficient conditions. Fatty acid analysis revealed that knockdown strains contained more 16 : 0 and 18 : 2 than wild-type species. However, the mechanisms responsible for this skewing of the fatty acid profile are unclear. These results demonstrate that disrupting lipid catabolism is also a feasible approach to improve TAG accumulation in microalgae without affecting growth rates or biomass production.

Most microalgae are obligate photoautotrophs and require light for their growth. The requirement for light to increase cell densities and production is the main objective and a limiting factor of their cultivation [48]. Heterotrophic growth in conventional fermenters can be a cost-effective alternative to photoautotrophic growth. Zaslavskaia *et al*. [143] performed a trophic conversion of *P. tricornutum* by introducing a gene encoding a glucose transporter and demonstrated that transgenic *P. tricornutum* could thrive on exogenous glucose without light, thereby becoming heterotrophic.

Recently, a similar approach has been employed to create a heterotrophic strain of *P. tricornutum* that also accumulates enhanced levels of DHA [144]. This was achieved by generating transgenic strains co-expressing Δ5-elongase from *O. tauri* with a glucose transporter from the moss *Physcomitrella patens*. This double transformant has the capacity to grow in the dark in liquid medium supplemented with glucose and accumulate substantial levels of omega-3 LC-PUFAs. The effects of glucose concentrations on growth and LC-PUFA production of wild-type and transformed strains cultivated in the light and dark were studied. The highest accumulation of omega-3 LC-PUFAs was observed in cultures grown under mixotrophic conditions in the presence of 1% glucose (up to 32.2% of total fatty acids). Both DHA and EPA were detected at high levels in the neutral lipids of transgenic cells grown under phototrophic conditions, averaging 36.5% and 23.6% of total fatty acids, respectively. This study demonstrates the potential for *P. tricornutum* to be developed as a viable commercial strain for both EPA and DHA production under mixo- and heterotrophic conditions.

## 4. Conclusion

During the past decade and a half, intense research has been performed and considerable progress made in the genetic engineering of diatoms. Based on recent advances, it is clear that rational metabolic engineering of microalgae has a promising future for designing strains with improved lipid accumulation and desired fatty acid content. It is likely that a combination of strategies will provide the most feasible approach to improve LC-PUFA production and accumulation in TAG without inhibiting growth rates. In particular, our ever-improving understanding of lipid metabolism, combined with the promise of synthetic biology approaches (design–build–test), increased automation and HTP analyses, have the capacity to provide a literally transformative shift in our ability to direct the biosynthesis of any chosen compound. Ultimately, a digital blueprint of diatom metabolism would be available which would be predictive for varied inputs and outputs in (e.g.) lipid biosynthesis. By such computationally supported approaches, the true promise of predictive biology to deliver useful outputs will be realized.

## Supplementary Material

Supplementary information

## Supplementary Material

References for Table 3

## Data accessibility

This article has no additional data.
