# Supplementary material for: Modulation of lipid biosynthesis by stress in diatoms
Source: Philos Trans R Soc Lond B Biol Sci. 2017 Jul 17;372(1728):20160407. doi: 10.1098/rstb.2016.0407 (PMC5516116; doi:10.1098/rstb.2016.0407)
Supplement: Supplementary information [file rstb20160407supp1.docx]

# Electronic Supplementary data S1 : Environmental factors modify lipid and fatty acid metabolism

**SD1.1 – Introduction**

Marine diatoms are known to be rich in PUFA and specifically in EPA [1]. In the diatom *P. tricornutum*, it has been shown that EPA is synthesized as an end-product of the PUFA synthesis through elongation and desaturation steps with multiple metabolic pathways involving classical omega-6 and omega-3 pathways [2]. In the *Fistulifera* sp. as well as in *P. tricornutum*, it has been proposed that the fatty acid desaturation pathway was distributed between the chloroplast and the endoplasmic reticulum compartments, the final step corresponding to the EPA synthesis localized in the ER compartment before being incorporated into the chloroplastic glycerolipids [3], [4]. In *P. tricornutum*, to make possible this synthesis, two enzymes has been proposed: the ERdelta-5FAD1 has been characterized and a ERdelta-5FAD2 has been predicted. These enzymes involve the 20:4 (Δ8,11,14,17) linked to phospholipids (FAD1) or betaine lipids (FAD2) as a substrate to produce EPA [4].

Initially, diatom cultures have been developed for an aquaculture use to feed larvae. Among the tested diatoms, *Chaetoceros calcitrans* was shown to be a good source of EPA for larval and adult filter feeders in this field [5]. It has been reported that the highest level of EPA was obtained during the exponential growth phase. Initially EPA is concentrated into the glycolipid fraction, that decreases during the logarithmic growth, to the benefit of the neutral lipid fraction.

**SD1.2 – Influence of carbon availability**

A carbon source is absolutely required for lipid and fatty acid accumulation. Indeed, without this source, independently of nutrient deprivation, biomass or lipid synthesis is not possible. Diatoms as photoautotroph eukaryotes can use inorganic carbon such as CO_2_ as carbon source using light as energy source, this participating to the reduction of the atmospheric CO_2_ [6] (Heydarizadeh et al. this volume). Bicarbonate has been shown to increase the lipid accumulation in diatoms such as *P. tricornutum* [7]. This effect does not result from bicarbonate addition per se but from the concomitant pH increase [8]. Using ^13^C labeling, an equal incorporation of ^13^C carbon issued from bicarbonate has been observed in the C16 fatty acids chains incorporated into triacylglycerols and phosphatidylcholine, indicating that *P. tricornutum* is able to assimilate similar amounts of carbon regardless the nitrate availability. This suggests that other internal lipid pool such as glycine betaine could be involved in the TG or PC synthesis [9].

Mixotrophy with glycerol as a carbon source and ammonium as a nitrogen source enhances growth of the diatom *P. tricornutum*. The periodic supplementation of culture medium with glycerol and ammonium chloride increased by a factor 10 the productivity of EPA when compared with photoautotrophically control conditions [10].

In *P. tricornutum*, high levels of CO_2_ (0.15%) induce an increase in long chain fatty acid levels, representing more than 16% of cell dry weight by comparison with around 7 and 8% obtained respectively with low (0.015%) and mid-CO_2_ (atmospheric, 0.035%) cultured cells [11]. The amounts of EPA are around 11.6% of cell dry weight (w/w) with high CO_2_ levels while they are around 5-7% under low and mid-CO_2_ conditions. However, the influence of CO_2_ levels depends on the studied diatoms species and specifically, concerning the amounts in EPA in the concerned diatom. Indeed, *P. tricornutum* is a rich EPA diatom and as above described, levels of CO_2_ enhances the EPA productivity. By comparison, *Chaetoceros muelleri* with low content in polyunsaturated fatty acids [12] has no change in its EPA content when the CO_2_ concentrations vary from 0.03 to 30% [13].

**SD1.3 – Influence of other nutrients**

In response to salt stress, diatoms are able to change their lipid and fatty acid compositions to modify their membrane permeability. Indeed, in the diatom *Nitzschia laevis*, Chen el al. [14],

testing different salt concentrations (from 10 to 30 g L^-1^), have shown that the highest levels of EPA were obtained with a salt concentration of 20 g L^-1^, in parallel with the highest level of galactolipids and phospholipids, EPA representing more than 70% of the total of fatty acids in these polar fractions. These changes in the unsaturation of membrane lipids were suggested to be related with a modification of membrane permeability and fluidity under salt concentration as an acclimation to salinity stress.

Among nitrogen sources that can be used by diatoms, NaNO_3_, NH_4_Cl and urea have been tested in *Cylindrotheca* species (fusiformis and closterium). Whatever the nitrogen source, EPA levels were not modified, even if the maximum of lipid accumulation was observed with the NH_4_Cl source and thus, whatever the studied species [15].

**SD1.4 – Environmental factors**

Environmental factors have different effects on lipid and fatty acid production in *P. tricornutum*. Salinity, nitrogen concentrations, light intensities or temperature can act differently on fatty acid composition and total fatty acid content. Indeed, with a salinity from 15 to 35 ppt or with different irradiance levels (50 to 150 µmol m^-2^ s^-1^), no modification is observed after 7 days of growth concerning the amounts of EPA. Decreased levels of EPA were observed under nitrogen limitation condition (1.24 mg L^-1^ *versus* 12.35 to 49.40 mg L^-1^) or increased temperature (25°C *versus* 15°C) [16].

**SD1.4.1 – Light**

In the marine diatom *Skeletonema costatum*, highest amounts of EPA are obtained at the end of the logarithmic growth phase and during the stationary phase under high light levels (saturating irradiance [17].

Quality of UV irradiance has different effect of fatty acid. Indeed, it has been reported that in benthic diatoms, even if modifications of fatty acid composition can occur after long-term (30 days) exposure to UV treatments, the UV-A exposure induces an increase in saturated fatty acids while the UV-B exposure increased the unsaturated fatty acid (EPA) levels. Concerning the lipid content, it increased after a short- term (6hrs) UV-B exposure [18].

**SD1.4.2 – Temperature**

Low levels of temperature have an impact on the EPA synthesis. Indeed, in the *P. tricornutum* strain, it has been shown that a decrease of culture temperature from 25°C to 10°C enhances the amounts of EPA with an increase of 120% by comparison with the control conditions [19]. Same results have been obtained with *Odontella aurita*, a diatom actually used in aquaculture but also in human nutrition, in which it has been shown increased levels in EPA, but also in DHA, at 8°C by comparison with 24 and 16°C [20].

**SD1.4.3 – Stress temperature – original results**

Material and Methods

*Microalgal culture, collection and storage*

In our standard culture conditions, axenic strain of  *P. tricornutum* UTEX 646 was cultivated at 22°C in flasks containing 500 mL of F/2 medium including silica and vitamins under an irradiance of 120 µmol PAR m^-2^ s^-1^ and a photoperiod of 14 h illumination and 10 h darkness.

At the initial day of culture, the concentration of microalgae was 10^5^ cells mL^-1^ of F/2 medium. Thermal stress was applied after 5 days of the growth during exponential growth phase. To perform this stress condition, one part of flask were shifted to 10°C for up to 8 days (stressed cultures) whereas others remained at 22°C (control).

For control and stressed cultures, cells were harvested after 1, 2, 4 and 8 days after temperature stress (respectively corresponding to 6, 7, 9 and 13 days of growth). Then, microalgae were separated from culture medium by soft centrifugation (1200 *x g*, 10 min, 4°C). The pellets were stored under N_2_ at 80°C, before being analyzed for pigment and lipids content. Each condition of culture was performed in triplicate. At the end of the experiment, cultures were still in exponential phase.

*Lipids quantification*

Microalgal pellets were freeze-dried before lipid extraction at -55°C and at 0.1 mbar, on a freeze-dryer equipped with a Christ Alpha 1-4 condenser. Then lipid were extracted with an Accelerated Solvent Extractor (DIONEX ASE 200) using a mixture of chloroform /methanol (proportion) at 40°C, 100 bars, 15 % of flush, 60s purge and 5mL cells. Three methods were successively used: (1) chloroform/methanol (25%/75% v/v), static time: 10min, (2) chloroform/methanol (75%/25% v/v), static time: 5min, (3) chloroform (100%), static time: 0min. A volume of 1% NaCl solution was added and the mixture separated into 2 phases. The lower layer containing lipids was collected and water removed by addition of dry sodium sulfate. These extracts were stored in the dark at -26°C.

Total fatty acid content was obtained by using a TH-10 Iatroscan. Operation conditions for the Iatroscan were 150 mL min^−1^ hydrogen flow, 2 L min^−1^ air flow, and 30 s/chromarod scan speed. Chromarods were activated by passing them through the FID scanner immediately before sample spotting and 1 𝜇L (≈20µg) of the sample solution (×5) was applied on each rod. The FID response wass measured and compared to a calibration standard (cod liver oil). The linearity of this calibration is verified from 5 to 35µg of lipid.

*Fatty acid analyses*

Total lipids were extracted with methanol/chloroform (2/1, v/v) after adding 200 μL of 2.8 g L^-1^ NaCl, a modified version of Bligh and Dyer's [21] method. Manual crushing (Dounce cells grinders) was coupled with ultrasonication step for 30min. Chloroform (1 mL) was added between the two ultrasonication steps in order to allow phase separation. The chloroform layer, containing the lipids, was collected and second extraction was carried out by adding 2 mL of chloroform to the remaining methanol/water phase. The solvents were removed by evaporating under vacuum, and all samples were dissolved in a known volume of chloroform. The lipid extracts were stored at 4°C under nitrogen gas (N_2_) to limit oxidization.

Total lipid extracts were fractionated on reversed phase silica gel columns (Sep-Pak Plus silica cartridges, Waters, France) after an activation step with 20 mL of methanol followed by 20 mL of chloroform. Neutral lipids were eluted using 20 mL of chloroform, polar lipids such as glycolipids were eluted with 40 mL of chloroform/methanol (5/1 v/v) (Sukenik et al. 1989) [22]. The different fractions obtained were dried by evaporating under a stream of N_2_. Thin-layer chromatography of the total lipid extract was carried out in order to check the purity of each fraction.

The solvent was then evaporated under N_2_, and the total fatty acids were extracted after saponifying with CH_3_OH-NaOH 0.5 M at 80°C for 20 min, according to the method of Slover and Lanza [23]. Total fatty acid methyl esters (FAMEs) were formed directly by treating the total extracts with boron trifluoride-methanol (BF_3_-MeOH, Sigma-Aldrich) at 80 °C for 20 min. The FAMEs were then extracted with iso-octane and 35% NaCl. All samples were analyzed with a FOCUS gas chromatography apparatus (Thermo Electron Corporation) equipped with a flame ionization detector, and a fused-silica capillary column (CP Sil-88 25 m×0.25 mm id capillary column). Samples were injected using an autoinjector AI 3000 (Thermo Electron Corporation). The injector and detector temperatures were 250 and 280°C, respectively, and the oven temperature was increased from 120 to 220°C at a rate of 6 °C.min^-1^. N_2_ was used as the carrier gas. Pure standards (Sigma-Aldrich) were used to identify the fatty acids by comparing the peak retention times of the samples and standards.

*Data statistical analysis*

All the results were analyzed by one-way analysis of variance (ANOVA). Post hoc analyses were then made by Student–Newman–Keuls (SNK) test to estimate the differences between treatment groups. Differences were considered significant at *p*<0.05. All statistics were performed with Statgraphic software.

**Table SD1**. Microalgal lipid content (% of biomas) and total lipid and galactolipid EPA levels (% molar of total fatty acids) for control (22°C) and stressed cultures (10°C) of *P. tricornutum* after 1, 2, 4 and 8 days of temperature stress.

After a one way ANOVA used in analysis of the influence of temperature stress, SNK multiple comparison test results are arranged in increasing order from left to right: a<b<c (*p*<0.05).

| **Stress** | **Day 1** | | **Day 2** | | **Day 4** | | **Day 8** | |
| --- | --- | --- | --- | --- | --- | --- | --- | --- |
| **Temperature** | **22°C** | **10°C** | **22°C** | **10°C** | **22°C** | **10°C** | **22°C** | **10°C** |
| **Lipid content** | 7.8 ± 1.3^a^ | 14.3 ± 1.8^c^ | 8.2 ± 0.6^a^ | 7.4 ± 0.9^a^ | 8.0 ± 2.1^a^ | 9.3 ± 1.1^ab^ | 10.7 ± 0.7^b^ | 9.4 ± 1.3^ab^ |
| **EPA level** |  | | | | | | | |
| Total lipids | 32.1 ± 1.6^a^ | 31.6 ± 2.3^a^ | 32.9 ± 1.4^a^ | 38.7 ± 2.1^b^ | 29.6 ± 2.9^a^ | 40.7 ± 0.2^b^ | 33.1 ± 1.6^a^ | 40.6 ± 3.0^b^ |
| Galactolipids | 40.2 ± 1.8^ab^ | 42.4 ± 2.2^b^ | 39.4 ± 1.1^ab^ | 50.9 ± 2.5^c^ | 39.1 ± 1.8^ab^ | 50.0 ± 0.21^c^ | 37.1 ± 4.3^a^ | 50.1 ± 6.2^c^ |

References

[1] Mimouni, V., Ulmann, L., Pasquet, V., Mathieu, M., Picot, L., Bougaran, G., Cadoret, J.-P., Morant-Manceau, A. & Schoefs, B. 2012 The potential of microalgae for the production of bioactive molecules of pharmaceutical interest. . *Current Pharmaceutical Biotechnology* **13**, 2733-2750.

[2] Arao, T. & Yamada, M. 1994 Biosynthesis of polyunsaturated fatty acids in the diatom *Phaeodactylum tricornutum*. *Phytochemistry* **35**, 1177-1181.

[3] Liang, Y., Maeda, Y., Sunaga, Y., Muto, M., Matsumoto, M., Yoshino, T. & Tanaka, T. 2013 Biosynthesis of polyunsaturated fatty acids in the oleaginous marine diatom *Fistulifera* sp Strain JPCC DA0580. *Marine Drugs* **11**, 5008-5023.

[4] Dolch, L.J. & Marechal, E. 2015 Inventory of Fatty Acid Desaturases in the Pennate Diatom *Phaeodactylum tricornutum*. *Marine Drugs* **13**, 1317-1339.

[5] Miller, M.R., Quek, S.Y., Staehler, K., Nalder, T. & Packer, M.A. 2014 Changes in oil content, lipid class and fatty acid composition of the microalga *Chaetoceros calcitrans* over different phases of batch culture. *Aquaculture Research* **45**, 1634-1647.

[6] Fields, M.W., Hise, A., Lhoman, E.J., Bell, T., Gardner, R.D., Corredor, L., Moll, K., Peyton, B.M., Characklis, G.W. & Gerlach, R. 2014 Sources and resources: importance of nutrients, resource allocation, and ecology in microalgal cultivation for lipid accumulation. *Applied Microbiology and Biotechnology* **98**, 4805-4816.

[7] Gardner, R.D., Cooksey, K.E., Mus, F., Macur, R., Moll, K., Eustance, E., Carlson, R.P., Gerlach, R., Fields, M.W. & Peyton, B.M. 2012 Use of sodium bicarbonate to stimulate triacylglycerol accumulation in the chlorophyte *Scenedesmus* sp. and the diatom *Phaeodactylum tricornutum*. *Journal of Applied Phycology* **24**, 1311-1320.

[8] Mus, F., Toussaint, J.-P., Cooksey, K.E., Fields, M.W., Gerlach, R., Peyton, B.M. & Carlson, R.P. 2013 Physiological and molecular analysis of carbon source supplementation and pH stress-induced lipid accumulation in the marine diatom *Phaeodactylum tricornutum*. *Applied Microbiology and Biotechnology* **97**, 3625-3642.

[9] Burrows, E.H., Benette, N.B., Carrieri, D., Dixon, J.L., Brinker, A., Frada, M., Baldassano, S.N., Falkowski, P.G. & Dismukes, G.C. 2012 Dynamics of lipid biosynthesis and redistribution in the marine diatom *Phaeodactylum tricornutum* under nitrate deprivation. *BioEnergy Research* **5**, 876-885.

[10] Ceron Garcia, M.C., Fernandez Sevilla, J.M., Aci‚n Fernandez, F.G., Molina Grima, E. & Garcia Camacho, F. 2000 Mixotrophic growth of Phaeodactylum tricornutum on glycerol: growth rate and fatty acid profile. *JOURNAL-OF-APPLIED-PHYCOLOGY* **12**, 239-248.

[11] Wu, S.C., Huang, A.Y., Zhang, B.Y., Huan, L., Zhao, P.P., Lin, A.P. & Wang, G.C. 2015 Enzyme activity highlights the importance of the oxidative pentose phosphate pathway in lipid accumulation and growth of *Phaeodactylum tricornutum* under CO_2_ concentration. *Biotechnology for Biofuels* **8**, 78.

[12] Hu, Q., Sommerfeld, M., Jarvis, E., Ghirardi, M., Posewitz, M., Seibert, M. & Darzins, A. 2008 Microalgal triacylglycerols as feedstocks for biofuel production; perspectives and advances. *PLANT-JOURNAL* **54**, 621-639.

[13] Wang, X.W., Liang, J.R., Luo, C.S., Chen, C.P. & Gao, Y.H. 2014 Biomass, total lipid production, and fatty acid composition of the marine diatom *Chaetoceros muelleri* in response to different CO_2_ levels. *Bioresource Technology* **16**, 124-130.

[14] Chen, G.Q., Jiang, Y. & Chen, F. 2008 Salt-induced alterations in lipid composition of diatom *Nitzschia laevis* (Bacillariophyceae) under heterotrophic culture condition. *Journal of Phycology* **44**, 1309-1314.

[15] Suman, K., Kiran, T., Devi, U.K. & Sarma, N.S. 2012 Culture medium optimization and lipid profiling of *Cylindrotheca*, a lipid- and polyunsaturated fatty acid-rich pennate diatom and potential source of eicosapentaenoic acid. *Botanica Marina* **55**, 289-299.

[16] Qiao, H.J., Cong, C., Sun, C.X., Li, B.S., Wang, J.Y. & Zhang, L.M. 2016 Effect of culture conditions on growth, fatty acid composition and DHA/EPA ratio of Phaeodactylum tricornutum. *Aquaculture* **452**, 311-317. (doi:10.1016/j.aquaculture.2015.11.011).

[17] Guihéneuf, F., Mimouni, V., Ulmann, L. & Tremblin, G. 2008 Environmental factors affecting growth and omega 3 fatty acid composition in *Skeletonema costatum*. The influences of irradiance and carbon source. *Diatom Research* **23**, 93-103.

[18] Scholz, B., Rua, A. & Liebezeit, G. 2014 Effects of UV radiation on five marine microphytobenthic Wadden sea diatoms, isolated from the Solthörn tidal flat (Lower Saxony, southern North Sea) - Part II: changes in carbohydrate, amino acid and fatty acid composition. *European Journal of Phycology* **49**, 97-114.

[19] Jiang, H. & Gao, K. 2004 Effects of lowering temperature during culture on the production of polyunsaturated fatty acids in the marine diatom *Phaeodactylum tricornutum* (Bacilariophyceae). *Journal of Phycology* **40**, 651-654.

[20] Pasquet, V., Ulmann, L., Mimouni, V., Guiheneuf, F., Jacquette, B., Morant-Manceau, A. & Tremblin, G. 2014 Fatty acids profile and temperature in the cultured marine diatom *Odontella aurita*. *Journal of Applied Phycology* **26**, 2265-2271.

[21] Bligh, E.G. & Dyer, W.J. 1959 A rapid method of total lipid extraction and purification. *Canadian Journal of Biochemical Physiology* **37**, 911-917.

[22] Sukenik, A., Carmeli, Y. & Berner, T. 1989 Regulation of fatty acid composition by irradiance level in the Eustigmatophyte *Nannochloropsis* sp. *Journal of Phycology* **25**, 686-692.

[23] Slover, H.F. & Lanza, E. 1979 Quantitative analysis of food fatty acids by capillary gas chromatography. *Journal of the American Oil Chemists Society* **56**, 933-943.
