# Supplementary material for: Modulation of lipid biosynthesis by stress in diatoms
Source: Philos Trans R Soc Lond B Biol Sci. 2017 Jul 17;372(1728):20160407. doi: 10.1098/rstb.2016.0407 (PMC5516116; doi:10.1098/rstb.2016.0407)
Supplement: References for Table 3 [file rstb20160407supp2.docx]

**References for Table 3**

Bromke MA, Giavalisco P, Willmitzer L, Hesse H (2013) Metabolic Analysis of Adaptation to Short-Term Changes in Culture Conditions of the Marine Diatom Thalassiosira pseudonana. Plos One 8 (6). doi:10.1371/journal.pone.0067340

Chen Y-C (2012) The biomass and total lipid content and composition of twelve species of marine diatoms cultured under various environments. Food Chemistry 131 (1):211-219. doi:10.1016/j.foodchem.2011.08.062

Chisti Y (2007) Biodiesel from microalgae. Biotechnology Advances 25 (3):294-306. doi:10.1016/j.biotechadv.2007.02.001

Cook O, Hildebrand M (2016) Enhancing LC-PUFA production in Thalassiosira pseudonana by overexpressing the endogenous fatty acid elongase genes. Journal of Applied Phycology 28 (2):897-905. doi:10.1007/s10811-015-0617-2

Daboussi F, Leduc S, Marechal A, Dubois G, Guyot V, Perez-Michaut C, Amato A, Falciatore A, Juillerat A, Beurdeley M, Voytas DF, Cavarec L, Duchateau P (2014) Genome engineering empowers the diatom Phaeodactylum tricornutum for biotechnology. Nature Communications 5. doi:10.1038/ncomms4831

Dunahay TG, Jarvis EE, Roessler PG (1995) Genetic transformation of the diatoms Cyclotella cryptica and Navicula saprophila. Journal of Phycology 31 (6):1004-1012. doi:10.1111/j.0022-3646.1995.01004.x

Gong Y, Guo X, Wan X, Liang Z, Jiang M (2011) Characterization of a novel thioesterase (PtTE) from Phaeodactylum tricornutum. Journal of Basic Microbiology 51 (6):666-672. doi:10.1002/jobm.201000520

Hamilton M, Haslam RP, Sayanova O, Napier JA (2015) METABOLIC ENGINEERING OF DIATOMS FOR THE ENHANCED PRODUCTION OF HIGH VALUE LIPIDS. European Journal of Phycology 50:38-38

Levitan O, Dinamarca J, Zelzion E, Lun DS, Guerra LT, Kim MK, Kim J, Van Mooy BAS, Bhattacharya D, Falkowski PG (2015) Remodeling of intermediate metabolism in the diatom Phaeodactylum tricornutum under nitrogen stress. Proceedings of the National Academy of Sciences of the United States of America 112 (2):412-417. doi:10.1073/pnas.1419818112

Ma Y-H, Wang X, Niu Y-F, Yang Z-K, Zhang M-H, Wang Z-M, Yang W-D, Liu J-S, Li H-Y (2014) Antisense knockdown of pyruvate dehydrogenase kinase promotes the neutral lipid accumulation in the diatom Phaeodactylum tricornutum. Microbial Cell Factories 13. doi:10.1186/s12934-014-0100-9

Matsumoto M, Sugiyama H, Maeda Y, Sato R, Tanaka T, Matsunaga T (2010) Marine Diatom, Navicula sp Strain JPCC DA0580 and Marine Green Alga, Chlorella sp Strain NKG400014 as Potential Sources for Biodiesel Production. Applied Biochemistry and Biotechnology 161 (1-8):483-490. doi:10.1007/s12010-009-8766-x

Niu Y-F, Zhang M-H, Li D-W, Yang W-D, Liu J-S, Bai W-B, Li H-Y (2013) Improvement of Neutral Lipid and Polyunsaturated Fatty Acid Biosynthesis by Overexpressing a Type 2 Diacylglycerol Acyltransferase in Marine Diatom Phaeodactylum tricornutum. Marine Drugs 11 (11):4558-4569. doi:10.3390/md11114558

Niu YF, Wang X, Hu DX, Balamurugan S, Li DW, Yang WD, Liu JS, Li HY (2016) Molecular characterization of a glycerol-3-phosphate acyltransferase reveals key features essential for triacylglycerol production in Phaeodactylum tricornutum. Biotechnology for Biofuels 9. doi:10.1186/s13068-016-0478-1

Peng K-T, Zheng C-N, Xue J, Chen X-Y, Yang W-D, Liu J-S, Bai W, Li H-Y (2014) Delta 5 Fatty Acid Desaturase Upregulates the Synthesis of Polyunsaturated Fatty Acids in the Marine Diatom Phaeodactylum tricornutum. Journal of Agricultural and Food Chemistry 62 (35):8773-8776. doi:10.1021/jf5031086

Radakovits R, Eduafo PM, Posewitz MC (2011) Genetic engineering of fatty acid chain length in Phaeodactylum tricornutum. Metabolic Engineering 13 (1):89-95. doi:10.1016/j.ymben.2010.10.003

Rodolfi L, Zittelli GC, Bassi N, Padovani G, Biondi N, Bonini G, Tredici MR (2009) Microalgae for Oil: Strain Selection, Induction of Lipid Synthesis and Outdoor Mass Cultivation in a Low-Cost Photobioreactor. Biotechnology and Bioengineering 102 (1):100-112. doi:10.1002/bit.22033

Satoh A, Ichii K, Matsumoto M, Kubota C, Nemoto M, Tanaka M, Yoshino T, Matsunaga T, Tanaka T (2013) A process design and productivity evaluation for oil production by indoor mass cultivation of a marine diatom, Fistulifera sp JPCC DA0580. Bioresource Technology 137:132-138. doi:10.1016/j.biortech.2013.03.087

Tonon T, Harvey D, Larson TR, Graham IA (2002) Long chain polyunsaturated fatty acid production and partitioning to triacylglycerols in four microalgae. Phytochemistry 61 (1):15-24. doi:10.1016/s0031-9422(02)00201-7

Trentacoste EM, Shrestha RP, Smith SR, Gle C, Hartmann AC, Hildebrand M, Gerwick WH (2013) Metabolic engineering of lipid catabolism increases microalgal lipid accumulation without compromising growth. Proceedings of the National Academy of Sciences of the United States of America 110 (49):19748-19753. doi:10.1073/pnas.1309299110

Wen ZY, Chen F (2003) Heterotrophic production of eicosapentaenoic acid by microalgae. Biotechnology Advances 21 (4):273-294. doi:10.1016/s0734-9750(03)00051-x

Xue J, Niu YF, Huang T, Yang WD, Liu JS, Li HY (2015) Genetic improvement of the microalga *Phaeodactylum tricornutum* for boosting neutral lipid accumulation. Metabolic Engineering 27:1-9

Yao Y, Lu Y, Peng K-T, Huang T, Niu Y-F, Xie W-H, Yang W-D, Liu J-S, Li H-Y (2014) Glycerol and neutral lipid production in the oleaginous marine diatom Phaeodactylum tricornutum promoted by overexpression of glycerol-3-phosphate dehydrogenase. Biotechnology for Biofuels 7. doi:10.1186/1754-6834-7-110

Yongmanitchai W, Ward OP (1991) GROWTH OF AND OMEGA-3-FATTY-ACID PRODUCTION BY PHAEODACTYLUM-TRICORNUTUM UNDER DIFFERENT CULTURE CONDITIONS. Applied and Environmental Microbiology 57 (2):419-425

Zhu BH, Shi HP, Yang GP, Lv NN, Yang M, Pan KH (2016) Silencing UDP-glucose pyrophosphorylase gene in Phaeodactylum tricornutum affects carbon allocation. New Biotechnology 33 (1):237-244. doi:10.1016/j.nbt.2015.06.003

Zhukova NV, Aizdaicher NA (1995) FATTY-ACID COMPOSITION OF 15 SPECIES OF MARINE MICROALGAE. Phytochemistry 39 (2):351-356. doi:10.1016/0031-9422(94)00913-e

Cook O, Hildebrand M (2016) Enhancing LC-PUFA production in Thalassiosira pseudonana by overexpressing the endogenous fatty acid elongase genes. Journal of Applied Phycology 28 (2):897-905. doi:10.1007/s10811-015-0617-2

Daboussi F, Leduc S, Marechal A, Dubois G, Guyot V, Perez-Michaut C, Amato A, Falciatore A, Juillerat A, Beurdeley M, Voytas DF, Cavarec L, Duchateau P (2014) Genome engineering empowers the diatom Phaeodactylum tricornutum for biotechnology. Nature Communications 5. doi:10.1038/ncomms4831

Dunahay TG, Jarvis EE, Roessler PG (1995) Genetic transformation of the diatoms Cyclotella cryptica and Navicula saprophila. Journal of Phycology 31 (6):1004-1012. doi:10.1111/j.0022-3646.1995.01004.x

Gong Y, Guo X, Wan X, Liang Z, Jiang M (2011) Characterization of a novel thioesterase (PtTE) from Phaeodactylum tricornutum. Journal of Basic Microbiology 51 (6):666-672. doi:10.1002/jobm.201000520

Hamilton M, Haslam RP, Sayanova O, Napier JA (2015) METABOLIC ENGINEERING OF DIATOMS FOR THE ENHANCED PRODUCTION OF HIGH VALUE LIPIDS. European Journal of Phycology 50:38-38

Levitan O, Dinamarca J, Zelzion E, Lun DS, Guerra LT, Kim MK, Kim J, Van Mooy BAS, Bhattacharya D, Falkowski PG (2015) Remodeling of intermediate metabolism in the diatom Phaeodactylum tricornutum under nitrogen stress. Proceedings of the National Academy of Sciences of the United States of America 112 (2):412-417. doi:10.1073/pnas.1419818112

Ma Y-H, Wang X, Niu Y-F, Yang Z-K, Zhang M-H, Wang Z-M, Yang W-D, Liu J-S, Li H-Y (2014) Antisense knockdown of pyruvate dehydrogenase kinase promotes the neutral lipid accumulation in the diatom Phaeodactylum tricornutum. Microbial Cell Factories 13. doi:10.1186/s12934-014-0100-9

Niu Y-F, Zhang M-H, Li D-W, Yang W-D, Liu J-S, Bai W-B, Li H-Y (2013) Improvement of Neutral Lipid and Polyunsaturated Fatty Acid Biosynthesis by Overexpressing a Type 2 Diacylglycerol Acyltransferase in Marine Diatom Phaeodactylum tricornutum. Marine Drugs 11 (11):4558-4569. doi:10.3390/md11114558

Niu YF, Wang X, Hu DX, Balamurugan S, Li DW, Yang WD, Liu JS, Li HY (2016) Molecular characterization of a glycerol-3-phosphate acyltransferase reveals key features essential for triacylglycerol production in Phaeodactylum tricornutum. Biotechnology for Biofuels 9. doi:10.1186/s13068-016-0478-1

Peng K-T, Zheng C-N, Xue J, Chen X-Y, Yang W-D, Liu J-S, Bai W, Li H-Y (2014) Delta 5 Fatty Acid Desaturase Upregulates the Synthesis of Polyunsaturated Fatty Acids in the Marine Diatom Phaeodactylum tricornutum. Journal of Agricultural and Food Chemistry 62 (35):8773-8776. doi:10.1021/jf5031086

Radakovits R, Eduafo PM, Posewitz MC (2011) Genetic engineering of fatty acid chain length in Phaeodactylum tricornutum. Metabolic Engineering 13 (1):89-95. doi:10.1016/j.ymben.2010.10.003

Trentacoste EM, Shrestha RP, Smith SR, Gle C, Hartmann AC, Hildebrand M, Gerwick WH (2013) Metabolic engineering of lipid catabolism increases microalgal lipid accumulation without compromising growth. Proceedings of the National Academy of Sciences of the United States of America 110 (49):19748-19753. doi:10.1073/pnas.1309299110

Xue J, Niu YF, Huang T, Yang WD, Liu JS, Li HY (2015) Genetic improvement of the microalga *Phaeodactylum tricornutum* for boosting neutral lipid accumulation. Metabolic Engineering 27:1-9

Yao Y, Lu Y, Peng K-T, Huang T, Niu Y-F, Xie W-H, Yang W-D, Liu J-S, Li H-Y (2014) Glycerol and neutral lipid production in the oleaginous marine diatom Phaeodactylum tricornutum promoted by overexpression of glycerol-3-phosphate dehydrogenase. Biotechnology for Biofuels 7. doi:10.1186/1754-6834-7-110

Zhu BH, Shi HP, Yang GP, Lv NN, Yang M, Pan KH (2016) Silencing UDP-glucose pyrophosphorylase gene in Phaeodactylum tricornutum affects carbon allocation. New Biotechnology 33 (1):237-244. doi:10.1016/j.nbt.2015.06.003

Bromke MA, Giavalisco P, Willmitzer L, Hesse H (2013) Metabolic Analysis of Adaptation to Short-Term Changes in Culture Conditions of the Marine Diatom Thalassiosira pseudonana. Plos One 8 (6). doi:10.1371/journal.pone.0067340

Chen Y-C (2012) The biomass and total lipid content and composition of twelve species of marine diatoms cultured under various environments. Food Chemistry 131 (1):211-219. doi:10.1016/j.foodchem.2011.08.062

Chisti Y (2007) Biodiesel from microalgae. Biotechnology Advances 25 (3):294-306. doi:10.1016/j.biotechadv.2007.02.001

Cook O, Hildebrand M (2016) Enhancing LC-PUFA production in Thalassiosira pseudonana by overexpressing the endogenous fatty acid elongase genes. Journal of Applied Phycology 28 (2):897-905. doi:10.1007/s10811-015-0617-2

Daboussi F, Leduc S, Marechal A, Dubois G, Guyot V, Perez-Michaut C, Amato A, Falciatore A, Juillerat A, Beurdeley M, Voytas DF, Cavarec L, Duchateau P (2014) Genome engineering empowers the diatom Phaeodactylum tricornutum for biotechnology. Nature Communications 5. doi:10.1038/ncomms4831

Dunahay TG, Jarvis EE, Roessler PG (1995) Genetic transformation of the diatoms Cyclotella cryptica and Navicula saprophila. Journal of Phycology 31 (6):1004-1012. doi:10.1111/j.0022-3646.1995.01004.x

Gong Y, Guo X, Wan X, Liang Z, Jiang M (2011) Characterization of a novel thioesterase (PtTE) from Phaeodactylum tricornutum. Journal of Basic Microbiology 51 (6):666-672. doi:10.1002/jobm.201000520

Hamilton M, Haslam RP, Sayanova O, Napier JA (2015) METABOLIC ENGINEERING OF DIATOMS FOR THE ENHANCED PRODUCTION OF HIGH VALUE LIPIDS. European Journal of Phycology 50:38-38

Levitan O, Dinamarca J, Zelzion E, Lun DS, Guerra LT, Kim MK, Kim J, Van Mooy BAS, Bhattacharya D, Falkowski PG (2015) Remodeling of intermediate metabolism in the diatom Phaeodactylum tricornutum under nitrogen stress. Proceedings of the National Academy of Sciences of the United States of America 112 (2):412-417. doi:10.1073/pnas.1419818112

Ma Y-H, Wang X, Niu Y-F, Yang Z-K, Zhang M-H, Wang Z-M, Yang W-D, Liu J-S, Li H-Y (2014) Antisense knockdown of pyruvate dehydrogenase kinase promotes the neutral lipid accumulation in the diatom Phaeodactylum tricornutum. Microbial Cell Factories 13. doi:10.1186/s12934-014-0100-9

Niu Y-F, Zhang M-H, Li D-W, Yang W-D, Liu J-S, Bai W-B, Li H-Y (2013) Improvement of Neutral Lipid and Polyunsaturated Fatty Acid Biosynthesis by Overexpressing a Type 2 Diacylglycerol Acyltransferase in Marine Diatom Phaeodactylum tricornutum. Marine Drugs 11 (11):4558-4569. doi:10.3390/md11114558

Niu YF, Wang X, Hu DX, Balamurugan S, Li DW, Yang WD, Liu JS, Li HY (2016) Molecular characterization of a glycerol-3-phosphate acyltransferase reveals key features essential for triacylglycerol production in Phaeodactylum tricornutum. Biotechnology for Biofuels 9. doi:10.1186/s13068-016-0478-1

Peng K-T, Zheng C-N, Xue J, Chen X-Y, Yang W-D, Liu J-S, Bai W, Li H-Y (2014) Delta 5 Fatty Acid Desaturase Upregulates the Synthesis of Polyunsaturated Fatty Acids in the Marine Diatom Phaeodactylum tricornutum. Journal of Agricultural and Food Chemistry 62 (35):8773-8776. doi:10.1021/jf5031086

Radakovits R, Eduafo PM, Posewitz MC (2011) Genetic engineering of fatty acid chain length in Phaeodactylum tricornutum. Metabolic Engineering 13 (1):89-95. doi:10.1016/j.ymben.2010.10.003

Rodolfi L, Zittelli GC, Bassi N, Padovani G, Biondi N, Bonini G, Tredici MR (2009) Microalgae for Oil: Strain Selection, Induction of Lipid Synthesis and Outdoor Mass Cultivation in a Low-Cost Photobioreactor. Biotechnology and Bioengineering 102 (1):100-112. doi:10.1002/bit.22033

Tonon T, Harvey D, Larson TR, Graham IA (2002) Long chain polyunsaturated fatty acid production and partitioning to triacylglycerols in four microalgae. Phytochemistry 61 (1):15-24. doi:10.1016/s0031-9422(02)00201-7

Trentacoste EM, Shrestha RP, Smith SR, Gle C, Hartmann AC, Hildebrand M, Gerwick WH (2013) Metabolic engineering of lipid catabolism increases microalgal lipid accumulation without compromising growth. Proceedings of the National Academy of Sciences of the United States of America 110 (49):19748-19753. doi:10.1073/pnas.1309299110

Wen ZY, Chen F (2003) Heterotrophic production of eicosapentaenoic acid by microalgae. Biotechnology Advances 21 (4):273-294. doi:10.1016/s0734-9750(03)00051-x

Xue J, Niu YF, Huang T, Yang WD, Liu JS, Li HY (2015) Genetic improvement of the microalga Phaeodactylum tricornutum for boosting neutral lipid accumulation. Metabolic Engineering 27:1-9. doi:10.1016/j.ymben.2014.10.002

Yao Y, Lu Y, Peng K-T, Huang T, Niu Y-F, Xie W-H, Yang W-D, Liu J-S, Li H-Y (2014) Glycerol and neutral lipid production in the oleaginous marine diatom Phaeodactylum tricornutum promoted by overexpression of glycerol-3-phosphate dehydrogenase. Biotechnology for Biofuels 7. doi:10.1186/1754-6834-7-110

Yongmanitchai W, Ward OP (1991) GROWTH OF AND OMEGA-3-FATTY-ACID PRODUCTION BY PHAEODACTYLUM-TRICORNUTUM UNDER DIFFERENT CULTURE CONDITIONS. Applied and Environmental Microbiology 57 (2):419-425

Zhu BH, Shi HP, Yang GP, Lv NN, Yang M, Pan KH (2016) Silencing UDP-glucose pyrophosphorylase gene in Phaeodactylum tricornutum affects carbon allocation. New Biotechnology 33 (1):237-244. doi:10.1016/j.nbt.2015.06.003

Zhukova NV, Aizdaicher NA (1995) FATTY-ACID COMPOSITION OF 15 SPECIES OF MARINE MICROALGAE. Phytochemistry 39 (2):351-356. doi:10.1016/0031-9422(94)00913-e

Cook O, Hildebrand M (2016) Enhancing LC-PUFA production in Thalassiosira pseudonana by overexpressing the endogenous fatty acid elongase genes. Journal of Applied Phycology 28 (2):897-905. doi:10.1007/s10811-015-0617-2

Daboussi F, Leduc S, Marechal A, Dubois G, Guyot V, Perez-Michaut C, Amato A, Falciatore A, Juillerat A, Beurdeley M, Voytas DF, Cavarec L, Duchateau P (2014) Genome engineering empowers the diatom Phaeodactylum tricornutum for biotechnology. Nature Communications 5. doi:10.1038/ncomms4831

Dunahay TG, Jarvis EE, Roessler PG (1995) Genetic transformation of the diatoms Cyclotella cryptica and Navicula saprophila. Journal of Phycology 31 (6):1004-1012. doi:10.1111/j.0022-3646.1995.01004.x

Gong Y, Guo X, Wan X, Liang Z, Jiang M (2011) Characterization of a novel thioesterase (PtTE) from Phaeodactylum tricornutum. Journal of Basic Microbiology 51 (6):666-672. doi:10.1002/jobm.201000520

Hamilton M, Haslam RP, Sayanova O, Napier JA (2015) METABOLIC ENGINEERING OF DIATOMS FOR THE ENHANCED PRODUCTION OF HIGH VALUE LIPIDS. European Journal of Phycology 50:38-38

Levitan O, Dinamarca J, Zelzion E, Lun DS, Guerra LT, Kim MK, Kim J, Van Mooy BAS, Bhattacharya D, Falkowski PG (2015) Remodeling of intermediate metabolism in the diatom Phaeodactylum tricornutum under nitrogen stress. Proceedings of the National Academy of Sciences of the United States of America 112 (2):412-417. doi:10.1073/pnas.1419818112

Ma Y-H, Wang X, Niu Y-F, Yang Z-K, Zhang M-H, Wang Z-M, Yang W-D, Liu J-S, Li H-Y (2014) Antisense knockdown of pyruvate dehydrogenase kinase promotes the neutral lipid accumulation in the diatom Phaeodactylum tricornutum. Microbial Cell Factories 13. doi:10.1186/s12934-014-0100-9

Niu Y-F, Zhang M-H, Li D-W, Yang W-D, Liu J-S, Bai W-B, Li H-Y (2013) Improvement of Neutral Lipid and Polyunsaturated Fatty Acid Biosynthesis by Overexpressing a Type 2 Diacylglycerol Acyltransferase in Marine Diatom Phaeodactylum tricornutum. Marine Drugs 11 (11):4558-4569. doi:10.3390/md11114558

Niu YF, Wang X, Hu DX, Balamurugan S, Li DW, Yang WD, Liu JS, Li HY (2016) Molecular characterization of a glycerol-3-phosphate acyltransferase reveals key features essential for triacylglycerol production in Phaeodactylum tricornutum. Biotechnology for Biofuels 9. doi:10.1186/s13068-016-0478-1

Peng K-T, Zheng C-N, Xue J, Chen X-Y, Yang W-D, Liu J-S, Bai W, Li H-Y (2014) Delta 5 Fatty Acid Desaturase Upregulates the Synthesis of Polyunsaturated Fatty Acids in the Marine Diatom Phaeodactylum tricornutum. Journal of Agricultural and Food Chemistry 62 (35):8773-8776. doi:10.1021/jf5031086

Radakovits R, Eduafo PM, Posewitz MC (2011) Genetic engineering of fatty acid chain length in Phaeodactylum tricornutum. Metabolic Engineering 13 (1):89-95. doi:10.1016/j.ymben.2010.10.003

Trentacoste EM, Shrestha RP, Smith SR, Gle C, Hartmann AC, Hildebrand M, Gerwick WH (2013) Metabolic engineering of lipid catabolism increases microalgal lipid accumulation without compromising growth. Proceedings of the National Academy of Sciences of the United States of America 110 (49):19748-19753. doi:10.1073/pnas.1309299110

Xue J, Niu YF, Huang T, Yang WD, Liu JS, Li HY (2015) Genetic improvement of the microalga Phaeodactylum tricornutum for boosting neutral lipid accumulation. Metabolic Engineering 27:1-9. doi:10.1016/j.ymben.2014.10.002

Yao Y, Lu Y, Peng K-T, Huang T, Niu Y-F, Xie W-H, Yang W-D, Liu J-S, Li H-Y (2014) Glycerol and neutral lipid production in the oleaginous marine diatom Phaeodactylum tricornutum promoted by overexpression of glycerol-3-phosphate dehydrogenase. Biotechnology for Biofuels 7. doi:10.1186/1754-6834-7-110

Zhu BH, Shi HP, Yang GP, Lv NN, Yang M, Pan KH (2016) Silencing UDP-glucose pyrophosphorylase gene in Phaeodactylum tricornutum affects carbon allocation. New Biotechnology 33 (1):237-244. doi:10.1016/j.nbt.2015.06.003
